# A Case Report of Cerebral Venous Thrombosis as a Complication of Coronavirus Disease 2019 in a Well-appearing Patient

**DOI:** 10.5811/cpcem.2020.11.49633

**Published:** 2020-12-07

**Authors:** Monica Logan, Kyle Leonard, Daniel Girzadas

**Affiliations:** Advocate Christ Medical Center, Department of Emergency Medicine, Oak Lawn, Illinois

**Keywords:** COVID-19, SARS-CoV-2, cerebral venous thrombosis, stroke, anticoagulation

## Abstract

**Introduction:**

While thrombotic complications of severe coronavirus disease 2019 (COVID-19) have been documented, the overall risk in non-critically ill cases of COVID-19 remains unknown.

**Case Report:**

We report a case of a previously healthy male patient who presented to the emergency department with headache and extremity paresthesia. The patient was diagnosed with cerebral venous thrombosis (CVT) and found to have a positive COVID-19 test. Inpatient anticoagulation was initiated, and symptoms had largely resolved at discharge.

**Conclusion:**

This case demonstrates the importance of considering thrombotic complications, such as CVT, even in well-appearing COVID-19 patients with no other risk factors for thromboembolic disease.

## INTRODUCTION

Severe acute respiratory syndrome coronavirus 2 (SARS-CoV-2) first emerged in Wuhan, China, in December 2019 as the cause of coronavirus disease 2019 (COVID-19). Since that time, COVID-19 has escalated into a global pandemic as declared by the World Health Organization. Although recognized primarily as a respiratory disease, a growing body of literature has highlighted the association between COVID-19 and significant thromboembolic complications.^1^,^2^ We report a case of a 34-year old male presenting with cerebral venous thrombosis (CVT) in the setting of subacute febrile respiratory illness who was found to be SARS-CoV-2 positive on admission.

Cerebral venous thrombosis is an uncommon cause of stroke affecting approximately 5/1,000,000 people annually, accounting for <1% of all strokes.^3^ CVT presents at an earlier age compared to other causes of stroke, typically less than 50 years of age, and affects women more than men at a rate of 3:1, likely due to gender-specific risk factors (pregnancy, postpartum period, oral contraceptive use).^4^ With successful diagnosis and treatment, CVT carries a relatively low overall mortality rate compared with arterial stroke but has a risk of intracranial hemorrhage and a significant incidence of cognitive impairment and difficulty returning to work.^5^

## CASE REPORT

A healthy 34-year-old male employed as a baggage handler at an international airport presented to the emergency department (ED) with a frontal headache, dizziness, left neck pain, as well as paresthesia and subjective weakness in the right upper and lower extremities. One month prior to this presentation, the patient had 10 days of fevers, dry cough, fatigue, diarrhea, anosmia, and ageusia. He had a known SARS-CoV-2 positive contact, but personally had two negative nasopharyngeal tests for SARS-CoV-2. On day 14 after symptom resolution, the patient had a forceful sneeze and sudden onset of a severe, generalized headache. Following the initial onset the headache became intermittent and would resolve with acetaminophen. The patient subsequently developed blurry vision, imbalance with ambulation, as well as numbness and tingling in his right upper and lower extremities. He decided to come to the ED after dropping objects from his right hand. On review of systems he denied any symptoms of fever, myalgia, nausea, vomiting, abdominal pain, chest pain, shortness of breath, or diarrhea within the prior week. He had no past medical history and denied smoking and drug use.

On physical examination the patient was well appearing with a normal body habitus. His vital signs were as follows: temperature 37.2 degrees Celsius; blood pressure 146/98 millimeters mercury; pulse 80 beats per minute (min); respiratory rate 16 breaths per min; and pulse oximetry 95 percent on room air. The cardiopulmonary examination was unremarkable, and he had normal distal pulses in all extremities. Examination of the ears, nose, and throat was unremarkable. His neurological examination, including full cranial nerve testing, strength, sensation, coordination, and ambulation, was without appreciable deficit. His National Institute of Health Stroke Scale (NIHSS) score was 0.

Laboratory evaluation was significant for a D-dimer quantitative level of 2.31 milligrams per liter (mg/L) fibrinogen-equivalent-units (reference range ≤ 0.5 mg/L). The complete blood count, electrolytes, blood glucose, liver enzymes, and kidney function were within normal limits. The initial differential diagnosis included concern for possible carotid or vertebral artery dissection based on headache, left neck pain, and contralateral extremity paresthesia following a forceful sneeze. The patient was sent for a computed tomography angiogram of the head and neck, which revealed absent enhancement and filling defects within the superior sagittal sinus, torcula, left transverse, and left sigmoid sinus, as well as the proximal left internal jugular vein, compatible with cerebral CVT. There was also a partial filling defect within the right transverse venous sinus compatible with partial thrombosis. At this initial ED visit the patient also had a positive rapid SARS-CoV-2 test as part of his routine admission screening. He was treated with intravenous heparin and admitted to the COVID-19 intensive care unit (ICU) with neurology and hematology services on consultation.

During his hospitalization the patient had magnetic resonance imaging, which confirmed presence of dural venous sinus thrombosis along with increased signal in the left posterior frontal and parietal lobes suggestive of a small subarachnoid hemorrhage vs cerebral spinal fluid effusion without acute infarct or midline shift. The hematology consultant ordered hypercoagulability testing that showed a negative factor V Leiden mutation, no activated protein C resistance, undetected Janus kinase-2 mutation, negative peripheral blood flow cytometry for paroxysmal nocturnal hemoglobinuria, negative beta-2 glycoprotein antibodies, and normal protein S activity, with anticardiolipin antibodies still pending. He was positive for circulating lupus anticoagulant although this sample was obtained after initiation of heparin. Lupus anticoagulant testing was to be repeated in a few months out of concern for a possible false-positive result.

CPC-EM Capsule *Pending*

What do we already know about this clinical entity?

Cerebral venous thrombosis is a rare neurovascular emergency that has been reported in patients with current or recent coronavirus disease 2019 (COVID-19).

What makes this presentation of disease reportable?

This is the first case report in the emergency medicine literature of a patient found to have cerebral venous thrombosis in the setting of COVID-19.

What is the major learning point?

Cerebral venous thrombosis is a documented complication of COVID-19 and can present in otherwise well-appearing patients with no other risk factors for thrombotic disease.

How might this improve emergency medicine practice?

The delay from initial viral illness to onset of thrombotic complication highlights special considerations in evaluating emergency department patients with recent COVID-19 or in those who have already recovered.

At the time of discharge the patient’s headache and paresthesia had resolved. He continued to have subjective visual changes as well as a feeling of imbalance. The patient was discharged home on warfarin with close hematology and neurology follow-up.

## DISCUSSION

Thrombotic disease has become a well-recognized complication of COVID-19, including deep vein thrombosis, pulmonary embolism, myocardial infarction, and stroke.^1^,^2^,^6^ The evidence for the increased incidence of thrombotic complications in COVID-19 patients has appropriately focused on the critically ill ICU population, whereas the incidence of thromboembolic complications in non-critically ill COVID-19 patients is considered to be lower.^7^,^8^

Multiple disease-specific mechanisms have been proposed to explain the increased risk of thrombosis seen in COVID-19. These include severe systemic inflammation leading to hypercoagulability, viral binding to angiotensin converting enzyme-2 receptors on endothelial cells, endothelial damage, and microvascular thrombosis, as well as patient-specific risk factors such as age, obesity, comorbid conditions, and inherited coagulopathic disease.^1^,^9^,^10^

In regard to CVT, there have not been any cases reported in the emergency medicine literature thus far, but there is a prior case series in the neuroradiology literature from a New York medical center describing three patients who developed CVT in the setting of COVID-19. All three patients presented with acute mental status change, had abnormal vital signs and/or abnormal neurologic findings and had multifocal pneumonia on chest imaging. All three of these cases were fatal.^11^ In contrast, our case involved a well-appearing patient who had a normal mental status, normal vital signs and a NIHSS score of 0. He had no identifiable risk factors for venous thromboembolism (VTE) by past medical or family history. Additionally, inpatient genetic testing did not identify any definitive inherited hypercoagulable condition.

ED laboratory testing was notable only for an elevated D-dimer level. There is growing evidence that D-dimer levels above 1 mg/L in patients with COVID-19 can help risk stratify patients for increased mortality and thromboembolic complications.^1^,^12^ Thus, emergency physicians may want to consider VTE, including CVT, in their differential diagnosis even in non-critically ill appearing COVID-19 patients who present with concerning symptoms. The additional finding of an elevated D-dimer level may prompt consideration of symptom-related diagnostic imaging even in the absence of other VTE risk factors.

The patient in our case had two prior negative nasopharyngeal SARS-CoV-2 tests prior to his ED visit. The sensitivity of these tests is not well defined. A recent article postulated that if a patient has a pretest probability of infection of 50% and the sensitivity of the SARS-CoV-2 test is 70%, a negative test still leaves the patient with a 23% chance of being infected.^13^ Until the testing characteristics of the SARS-CoV-2 test are better defined or improved, emergency physicians will need to continue to consider the possibility of Covid-19 infection and related complications, even when caring for symptomatic patients with a negative SARS-CoV-2 test.

Furthermore, the time lapse between the initial viral illness to the development of a serious thrombotic complication in our patient raises additional concerns. Proposed guidelines from both the American Society of Hematology and the American College of Chest Physicians currently recommend initiation of medical thromboprophylaxis only for hospitalized patients in the absence of contraindication.^1^,^14^,^15^ Additional expert consensus has also recommended consideration for further short-term thromboprophylaxis for COVID-19 patients who have been discharged from the hospital.^16^ As there are no current recommendations for thromboprophylaxis in patients not requiring hospitalization, it may be necessary to consider VTE, including CVT, in non-previously hospitalized patients who present with concerning symptoms weeks to months following their recovery.

## CONCLUSION

Based on prior literature, clinicians anticipate thrombotic complications in critically ill patients infected with SARS-CoV-2.^1^,^2^ A time lapse of 7–14 days or more between initial infection and development of serious thrombotic complications has been reported.^2^,^8^ The risk of thrombotic complications in non-critically ill COVID-19 patients is thought to be lower.^7^,^8^ Our case demonstrates that thrombotic complications such as CVT can occur even in well-appearing, ambulatory COVID-19 patients who present with concerning symptoms.

## References

[1] Kollias A, Kyriakoulis KG, Dimakakos E (2020). Thromboembolic risk and anticoagulant therapy in COVID-19 patients: emerging evidence and call for action. Br J Haematol.

[2] Klok FA, Kruip MJHA, van der Meer NJM (2020). Confirmation of the high cumulative incidence of thrombotic complications in critically ill ICU patients with COVID-19: an updated analysis. Thromb Res.

[3] Mehta A, Danesh J, Kuruvilla D (2019). Cerebral venous thrombosis headache. Curr Pain Headache Rep.

[4] Dmytriw AA, Song JSA, Yu E (2018). Cerebral venous thrombosis: state of the art diagnosis and management. Neuroradiology.

[5] Shakibajahromi B, Borhani-Haghighi A, Ghaedian M (2019). Early, delayed, and expanded intracranial hemorrhage in cerebral venous thrombosis. Acta Neurol Scand.

[6] Tal S, Spectre G, Kornowski R (2020). Venous thromboembolism complicated with COVID-19: What do we know so far?. Acta Haematol.

[7] Middeldorp S, Coppens M, van Haaps TF (2020). Incidence of venous thromboembolism in hospitalized patients with COVID-19. J Thromb Haemost.

[8] Al-Ani F, Chehade S, Lazo-Langner A (2020). Thrombosis risk associated with COVID-19 infection. A scoping review. Thromb Res.

[9] Sardu C, Gambardella J, Morelli MB (2020). Hypertension, thrombosis, kidney failure, and diabetes: Is COVID-19 an endothelial disease? A comprehensive evaluation of clinical and basic evidence. J Clin Med.

[10] Hemasian H, Ansari B (2020). First case of Covid-19 presented with cerebral venous thrombosis: a rare and dreaded case. Rev Neurol (Paris).

[11] Cavalcanti DD, Raz E, Shapiro M (2020). Cerebral venous thrombosis associated with COVID-19. AJNR Am J Neuroradiol.

[12] Artifoni M, Danic G, Gautier G (2020). Systematic assessment of venous thromboembolism in COVID-19 patients receiving thromboprophylaxis: incidence and role of D-dimer as predictive factors. J Thromb Thrombolysis.

[13] Woloshin S, Patel N, Kesselheim AS (2020). False negative tests for SARS-CoV-2 infection: challenges and implications. N Engl J Med.

[14] Barnes GD, Burnett A, Allen A (2020). Thromboembolism and anticoagulant therapy during the COVID-19 pandemic: interim clinical guidance from the anticoagulation forum. J Thromb Thrombolysis.

[15] Moores LK, Tritschler T, Brosnahan S (2020). Prevention, diagnosis, and treatment of VTE in patients with coronavirus disease 2019: CHEST Guideline and Expert Panel Report. Chest.

[16] Zhai Z, Li C, Chen Y (2020). Prevention and treatment of venous thromboembolism associated with coronavirus disease 2019 infection: a consensus statement before guidelines. Thromb Haemost.

